# Implementation of the WHO hand hygiene strategy in Faranah regional hospital, Guinea

**DOI:** 10.1186/s13756-020-00723-8

**Published:** 2020-05-14

**Authors:** S. A. Müller, A. O. K. Diallo, R. Wood, M. Bayo, T. Eckmanns, O. Tounkara, M. Arvand, M. Diallo, M. Borchert

**Affiliations:** 1 Charité – Universitätsmedizin Berlin, Institute of Tropical Medicine and International Health, Berlin, Germany; 2 Centre for International Health Protection, Robert Koch Institute, Berlin, Germany; 3 Faranah Regional Hospital, Faranah, Guinea; 4 Deutsche Gesellschaft für Internationale Zusammenarbeit (GIZ), Conakry, Guinea; 5 Unit for healthcare-associated Infections, Surveillance of Antibiotic Resistance and Consumption, Robert Koch Institute, Berlin, Germany; 6 Unit for Hospital Hygiene, Infection Prevention and Control, Robert Koch Institute, Berlin, Germany

**Keywords:** Hand hygiene, WHO multimodal strategy, First WHO global patient safety challenge, 5 moments, Clean care is safer care, Clean hands, Healthcare-associated infections, Nosocomial infections, Local disinfectant production, Guinea

## Abstract

**Background:**

Healthcare-associated infections are the most frequent adverse events in healthcare worldwide, with limited available evidence suggesting highest burden in resource-limited settings. Recent Ebola epidemics emphasize the disastrous impact that spread of infectious agents within healthcare facilities can have, accentuating the need for improvement of infection control practices. Hand hygiene (HH) measures are considered to be the most effective tool to prevent healthcare-associated infections. However, HH knowledge and compliance are low, especially in vulnerable settings such as Guinea.

The aim of PASQUALE (Partnership to Improve Patient Safety and Quality of Care) was to assess knowledge and compliance with HH and improve HH by incorporating the WHO HH Strategy within the Faranah Regional Hospital (FRH), Guinea.

**Methods:**

In a participatory approach, a team of FRH staff and leadership was invited to identify priorities of the hospital prior to the start of PASQUALE. The local hygiene committee was empowered to increase its activities and take ownership of the HH improvement strategy. A baseline assessment of knowledge, perception and compliance was performed months before the intervention. The main intervention consisted of local alcohol-based-hand-rub (ABHR) production, with final product efficacy testing, in conjunction with a training adapted to the needs identified in the baseline assessment. A follow-up assessment was conducted directly after the training. Effectiveness of the intervention was assessed via uncontrolled before-and-after comparison.

**Results:**

Baseline knowledge score (13.0/25) showed a significant increase to 19.0/25 in follow-up. Baseline-Compliance was 23.7% and increased significantly to 71.5% in follow-up. Compliance rose significantly across all professional groups except for midwifes and in all indications for HH, with the largest in the indication “Before aseptic tasks”. The increase in compliance was associated with the intervention and remained significant after adjusting for confounders. The local pharmacy successfully supplies the entire hospital. The local supply resulted in a ten-fold increase of monthly hospital disinfectant consumption.

**Conclusion:**

The WHO HH strategy is an adaptable and effective method to improve HH knowledge and compliance in a resource-limited setting. Local production is a feasible method for providing self-sufficient supply of ABHR to regional hospitals like the FRH. Participatory approaches like hygiene committee ownership builds confidence of sustainability.

## Background

Healthcare-associated infections (HAI) are the most frequent adverse events in healthcare worldwide and therefore a major threat to patient safety [1]. Limited available evidence suggests highest burden with prevalence up to 15.5% in resource-limited settings [2–4]. A recent study from Guinea reports an even higher prevalence of HAIs of up to 20% [5]. The International Nosocomial Infection Control Consortium stated in 2008 that not only the risk of healthcare-associated infections (HAI) is higher in developing countries, but also its impact on patients and health systems [6]. A systematic review has shown that 35–55% of HAI are preventable [7].

Recent epidemics in low and middle income countries, such as the Ebola Virus Disease outbreak in West Africa, emphasized the disastrous impact that the spread of infectious agents within healthcare facilities can have. During this outbreak 199 healthcare workers (HCW) were infected within Guinean healthcare facilities [8]. High healthcare-associated infection rates accentuate the need for improvement of infection control practices [3].

The World Health Organization (WHO) considers hand hygiene (HH) measures to be the most effective tool to prevent HAI [2, 9, 10]. However, current studies have found that HH knowledge and compliance are especially low in limited-resource settings such as East and West Sub-Saharan Africa [11–14].

Therefore, the objectives of this study were to assess knowledge, perception, and compliance with HH, improve HH and strengthen hospital performance by incorporating the WHO HH Strategy including the local production of alcohol-based hand-rub (ABHR) within the Faranah Regional Hospital (FRH).

## Methods

### Study setting

The study was carried out in the Faranah Regional Hospital (FRH), in Guinea, a partner hospital of the Robert Koch Institute, Berlin, Germany. The FRH is a governmental reference hospital for a population of 300,000 inhabitants with an adult literacy rate of approximately 32.0% [15]. The hospital employs 91 healthcare and administrative staff-members and is comprised of 16 wards, including surgery, laboratory and, in aftermath of the Ebola Virus Disease epidemic from 2013 to 2016, an isolation ward.

The study was conducted as part of the PASQUALE (Partnership to Improve Patient Safety and Quality of Care) project and funded by the GIZ ESTHER Alliance (*Ensemble pour une Solidarité Thérapeutique Hospitalière en Réseau*). The PASQUALE project responds to the first WHO Global Patient Safety Challenge: “Clean Care is Safer Care” [16]. Ethics approval was obtained from the *Comité National d’Ethique pour la Recherche en Santé*, Guinea.

### Study design

The study was conducted to assess feasibility and effectiveness of the WHO HH improvement strategy in this low-resource setting. All wards and currently employed HCWs (not including administrative or cleaning staff) were invited to participate. Activities consisted of four phases: preparatory phase, pre-intervention evaluation, intervention and post-intervention evaluation.

#### Phase 0: preparatory phase

In December 2017, a needs assessment was conducted together with the FRH staff and leadership. In this participatory assessment, it was decided to focus on HH, water supply and sterilization. Moreover, a qualitative research study on HH was conducted to gain further insight, the results of which are planned to be published separately.

#### Phase I: pre-intervention evaluation

In January 2018, a baseline assessment took place inviting all currently employed HCWs to participate. This baseline assessment included the surveys and questionnaires on ward infrastructure, HCWs’ perception on HH, HH knowledge, and an observation of HH practices, using the validated WHO tools [17]. HH practices were assessed using the WHO “My 5 Moments for Hand Hygiene” approach including the indication 1) before touching a patient, 2) before clean/aseptic procedures, 3) after body fluid exposure risk, 4) after touching a patient and 5) after touching patient surroundings [18]. The knowledge questionnaire included questions such as “What is the most frequent source of germs responsible for healthcare-associated infections”, “Which of the following HH actions prevents transmission of germs to the patient”, “What is the minimal time needed for ABHR to kill most germs on your hands” and “Which type of HH method is required in the following situations ( …)” . The perception questionnaires focused on the five core elements of the WHO HH strategy (system change, education, observation and feedback, reminders in the workplace and patient safety climate) with questions like “What is the effectiveness of HH in preventing healthcare-associated infection” and “( …) how effective would the following actions be to improve HH permanently in your institution ( …)” [19]. When participants required clarification of language or help with Likert scales, support was available by members of the research team. Each participant received an identification number that was planned to be used instead of their names for pairing baseline and follow-up data on HH knowledge and perception. The direct observations were carried out by trained researchers from the PASQUALE project during day shifts at random times, without prior announcement. HH indications and opportunities were recorded throughout the observation. A priority rule was applied to ensure that only one indication was associated with each opportunity. This rule specified a hierarchy for simultaneously occurring indications as follows: before aseptic/clean procedure > after body fluid exposure risk > after touching a patient > before touching a patient > after touching the patient surrounding [20].

#### Phase II: intervention

December 2018, a tailored workshop was conducted. This training was adapted to potential improvement points of HH knowledge and practice identified in the pre-intervention evaluation during phase I. The training was held on three occasions as a 1 day workshop for 24 participants at a time. As such, every HCW had the opportunity to participate without interrupting routine hospital functions.

Local production of ABHR was reintroduced in a designated manufacturing room of the hospital pharmacy with four batches (10 L each) per month. This production schedule was based on a national estimate for hospital-wide ABHR needs [21]. Production of ABHR was initially introduced by WHO in 2016, but manufacturing was not sustainable due to supply issues such as access to hydrogen peroxide and peroxide test strips. These challenges were overcome by fostering collaborations with experienced Nongovernmental Organizations, such as Expertise France, and local private suppliers. The ABHR was subsequently produced following “Formulation 1” from WHO guidelines. This formulation specifies usage of ethanol 96%, hydrogen peroxide 3%, glycerol 98% and boiled, cold water [19, 22]. To apply the learnt hygiene measures every HCW received a pocket bottle of 100 ml ABHR and every ward or consultation room a bottle of 500 ml ABHR. Bottle labeling included the instruction “apply a palmful (3ml), cover all surfaces of the hands, rub hands until dry”. Upon request of the partner in Faranah who wished to be reassured about the efficacy of the locally produced ABHR, a partial efficacy testing was carried out in laboratories of the division for Hospital Hygiene, Infection Prevention and Control at the Robert Koch Institute. The efficacy testing was performed as suspension test according to the European Norm DIN EN 13727 using *Enterococcus hirae* as test organism. Further support for the local pharmacy is ongoing and south-south information exchange is being fostered between the FRH and the other PASQUALE Project partner *Centre Hospitalier Universitaire* in Bouaké, Côte d’Ivoire. A conjunct ABHR production training was held in Bouaké with assistance of the FRH pharmacists and PASQUALE team in June 2019.

As part of the participatory approach a local coordinator was given the responsibility of fostering project work within the FRH by conducting observations, regular HH reminding sessions during staff meetings and promotions of the locally produced ABHR.

#### Phase III: post-intervention evaluation

During December 2018 to March 2019, the assessment was repeated following the same methodology as in the pre-intervention evaluation in Phase I, with the perception survey containing one additional post-intervention part asking about perceived effects of the intervention.

Furthermore, production of ABHR was monitored and consumption of ABHR was tracked 6 months before (July to December 2018) and after the intervention (January to June 2019).

### Statistical analysis

All data was entered in WHO preprogrammed Epi Info data templates and analyzed using Stata 15.2 (StataCorp LLC, College Station, Texas, USA). For HH knowledge questionnaire responses, a score was calculated equaling the number of correct answers (maximum score 25 points). The scores were summarized as medians and interquartile ranges. Since pairing was only possible for half of the study population (30/62), a sensitivity analysis comparing paired and unpaired Wilcoxon rank-sum tests was performed. As conclusion of both tests was the same (data not shown), only results of the unpaired Wilcoxon rank-sum test are presented in this paper. Two-tailed *p*-values less than 0.05 were considered to be statistically significant.

HH perceptions on the five components of the WHO HH Strategy were assessed in baseline and follow-up questionnaires. Additional post-intervention perception questions were reported as the total number and percentage of follow-up respondents answering “seven” on a seven-point Likert scale, with one equaling “not effective” and seven “very effective”.

HH compliance was calculated as the number of HH actions performed divided by the number of all opportunities requiring HH according the WHO 5 Moments of HH. Compliance at baseline and follow-up was compared using χ^2^ tests, by wards and by professional categories. Multiple linear regression was performed to assess the association between the intervention and knowledge score, exploring the confounding effect of gender, age group, profession and ward. Multivariable logistic regression was performed with pre/post-intervention period as the main independent variable and compliance as the primary outcome. Confounders proposed in the literature “type of ward”, “hand hygiene indication” and “professional category” were included in the initial logistic regression model and maintained there if the crude OR differed substantially from the adjusted one. Consequently, the confounders “hand hygiene indication” and “professional category” were included in the final model.

As most healthcare professionals had more than one HH opportunity, the observations were not independent. For confidentiality purposes, and following the WHO multimodal HH Improvement Strategy observation form, HCWs were not identified during observation. To account for this lack of independence a design effect of two was assumed and accounted for by doubling the standard error (22); this approach has been used before in a similar study [11, 23].

## Results

### Study-population

A total of 62 out of 74 HCWs (54.8% female) participated in the baseline and 72 out of 84 (58.3% female) in the follow-up assessment. HCWs were categorized into five professional groups, with “Nurse” and “Other” being the largest. The professional group “Other” is comprised primarily of auxiliary nurses and medical students. All main units were categorized separately, with “Other” being comprised of smaller wards such as ophthalmology, odontology and the laboratory. Despite its size, CTEpi (*Centre de Traitement des Epidémies*), is also displayed separately as it plays an important role in infection prevention and control (Table 1).

**Table 1:** Study population

			Baseline N (%)	Follow-up N (%)
Number of Respondents			62	72
Respondents by Profession				
Medical doctor			12 (19.4)	11 (15.3)
Nurse			20 (32.3)	20 (27.8)
Midwife			7 (11.3)	7 (9.7)
Technician			9 (14.5)	7 (9.7)
Others			14 (22.5)	27 (37.5)
Respondents by Unit				
Internal Medicine			9 (14.5)	7 (9.7)
Surgery			8 (12.9)	11 (15.3)
Emergency			10 (16.1)	10 (13.9)
Obstetrics			11 (17.7)	11 (15.3)
Paediatrics			4 (6.4)	10 (13.9)
CTEpi			5 (8.1)	2 (2.8)
Others			15 (24.2)	21 (29.2)

### Hand hygiene knowledge

A majority (88.7%) of baseline respondents reported having had previous training in HH within the last 3 years. The median knowledge score at baseline was 13.0 (IQR 11.0–15.0) and increased to 19.0 (IQR 17.0–21.5) out of 25 after intervention (*p* < 0.001). Knowledge improved significantly across all professional categories except for “Technicians” and all wards except for CTEpi, which had borderline significance. No significant differences between wards or professions could be shown. However, the professional group of “Medical doctor” and “Nurse” showed the largest improvement with a 7.5 point increase. Knowledge also rose across all wards with greatest observed development in “CTEpi”, followed by “Internal Medicine” and “Obstetrics”. The lowest increase was observed for the “Emergencies” ward (Table 2). Multiple linear regression showed that no substantial confounding by gender, age group, profession, or ward was present in the association between intervention and knowledge score.

**Table 2:** Median hand hygiene knowledge score (IQR), Regional Hospital Faranah; maximum score: 25

	Baseline	Follow-up	P*
Overall Knowledge Score	13.0 (11.0–15.0)	19.0 (17.0–21.5)	< 0.001
By professional categories			
Medical doctor	13.5 (12.0–15.0)	21.0 (17.0–24.0)	0.002
Nurse	11.5 (9.5–14.0)	19.0 (16.5–21.0)	< 0.001
Midwife	13.0 (11.0–14.0)	17.0 (17.0–20.0)	0.012
Technician	13.0 (12.0–14.0)	15.0 (12.0–22.0)	0.310
Other	15.0 (13.0–17.0)	19.0 (18.0–21.0)	< 0.001
By ward			
Internal Medicine	14.0 (11.0–14.0)	21.0 (17.0–23.0)	0.014
Surgery	13.5 (11.5–15.0)	18.0 (15.0–21.0)	0.003
Emergencies	13.0 (9.0–13.0)	17.0 (15.0–19.0)	0.005
Obstetrics	13.0 (11.0–14.0)	20.0 (17.0–23.0)	< 0.001
Paediatrics	12.0 (9.0–15.5)	18.5 (18.0–22.0)	0.010
CTEpi	12.0 (11.0–13.0)	21.5 (19.0–24.0)	0.053
Others	14.0 (13.0–17.0)	19.0 (15.0–22.0)	0.005

*obtained from Wilcoxon rank-sum test

### Hand hygiene perception

Perceived impact of the five core elements was high in baseline and follow-up showing positive reception of the WHO HH strategy. Over 85% of respondents considered HH effectiveness to be “high” or “very high” in both baseline and follow-up. Self-evaluated compliance revealed elevated estimates (99% in baseline, 80% in follow-up, respectively) but was not in accordance with results of compliance observation (Table 4). An additional section in follow-up questionnaires showed that perception of the intervention and its beneficial impact were highly positive (Table 3).

**Table 3:** HCWs’ perception about impact of intervention

	N (%)^a^
Has the use of ABHR made HH easier to practice in your daily work?	57 (90.5)
Is the use of ABHR well tolerated by your hands?	57 (89.1)
Did knowing the results of HH observation in your ward help you to improve your HH practices?	54 (84.4)
Has the fact of being observed made you paying more attention to your HH practices?	51 (82.3)
Were the educational activities that you participated in important to improve your HH practices?	56 (87.5)
Has the improvement of the safety climate (…) helped you personally to improve your HH practices?	49 (76.6)
Has your awareness of your role in preventing HAIs by improving your HH practices increased during the current HH promotional campaign?	55 (84.6)

^a^results are shown as number of respondents out of 72 selecting seven on a seven-point Likert scale, indicating a fully affirmative answer

### Hand hygiene compliance

During 34 sessions of observation, 941 occasions requiring HH were observed (384 at baseline, 557 during follow-up). The overall baseline compliance was 23.7%. The professional group “Nurse” had the highest observed number of HH opportunities but a baseline compliance of 5.3%. The “Medical doctor” group had the second highest number of HH opportunities and the highest baseline compliance of 51.7%. The follow-up assessment found nurses and medical doctors to still have highest numbers in HH opportunities (over 75% of all opportunities) with both groups showing a significant compliance increase of 43.3 and 34.0 percentage points, respectively. This substantial improvement is in accordance with results from the knowledge questionnaire (Table 2). All other professional groups, except midwives, also presented a significant compliance rise, and the overall compliance at follow-up increased by 47.8 percentage points (from 23.7 to 71.5%).

Compliance also rose significantly across all indications, with the highest improvement (from 11.4 to 90.0%) in the category “Before aseptic tasks”. Observation of this indication prior to the intervention showed HCWs using gloves rather than taking HH actions. The indication with the highest number of HH opportunities, “After contact with patient surroundings”, had the lowest compliance in both baseline and follow-up (2.1 and 50.0% respectively) (Table 4).

**Table 4:** Hand Hygiene Compliance at Baseline and Follow-up, Faranah Regional Hospital, Guinea

Variable	Baseline			Follow-up			P**
	No. of HH actions	No. (%) of HH opportunities	Compliance, % (95% CI)^a^	No. of HH actions	No. (%) of HH opportunities	Compliance, % (95% CI)^a^	
Overall	91	384	23.7 (15.2–32.2)	398	557	71.5 (63.9–79.0)	< 0.001
Professional category							
Medical Doctors	76	147 (38.3)	51.7 (35.5–67.9)	203	237 (42.5)	85.7 (76.7–94.6)	< 0.001
Nurse	8	151 (39.3)	5.3 (−1.9–12.5)	90	185 (33.2)	48.6 (34.2–63.1)	< 0.001
Midwife	1	28 (7.3)	3.6 (−10.4–17.6)	0	14 (2.5)	0	0.610
Technician	6	34 (8.9)	17.6 (−8.4–24.8)	49	56 (10.1)	87.5 (70.0–105.0)	< 0.001
Other	0	24 (6.3)	0 (0)	56	65 (11.7)	86.2 (69.2–103.6)	< 0.001
Indication							
Before patient contact	42	120 (31.3)	35.0 (17.9–52.1)	109	133 (23.9)	82.0 (68.8–95.1)	< 0.001
Before aseptic task	5	44 (11.5)	11.4 (−7.6–30.3)	36	40 (7.2)	90.0 (71.1–108.8)	< 0.001
After body fluid exposure risk	7	21 (5.5)	33.3 (−8.0–74.7)	35	39 (7.0)	89.7 (70.5–109.0)	0.001
After patient contact	35	102 (26.6)	34.3 (16.0–53.8)	109	127 (22.8)	85.8 (73.6–98.0)	< 0.001
After contact with patient surroundings	2	97 (25.3)	2.1 (−3.6–7.7)	109	218 (39.1)	50.0 (36.7–63.3)	< 0.001

^a^width of CI adjusted for lack of independence by inflating standard error by a factor of 2

** determined by χ^2^ test with standard error corrected by factor 2 to adjust for lack of independence

In the multivariable analysis, the increase in compliance was associated with the intervention (crude OR 6.67; 95% CI 3.87–11.47; *p* < 0.001). This association became stronger and remained significant after adjusting for confounders (adjusted OR 16.40; 95% CI 7.40–36.35; *p* < 0.001).

### Production and consumption of ABHR

Local production in the hospital pharmacy was relaunched in December 2018 with regularly scheduled 10 L batches four times a month. The cost of raw materials for local ABHR production fluctuated between 0.56€ per 100 ml, to 0.10€ during project time depending on market import prices. Average monthly ABHR consumption for the entire hospital increased post-intervention by a factor of 12.7 from 2.2 L in baseline to 28.0 L in follow-up.

Efficacy testing of the locally produced ABHR revealed ≥5 log10 reduction of *E. hirae* in three independent experiments, thus fulfilling the requirements of the European Norm DIN EN 13727 for this test organism.

## Discussion

To our knowledge PASQUALE is the first study implementing and evaluating the WHO multimodal HH Strategy in Guinea, a country affected by the 2014 Ebola Virus Disease outbreak. This study took place in the regional hospital of Faranah, a healthcare facility lacking access to running water, reliable electricity and other basic infrastructural components such as the WHO recommended sink: bed ratio [24].

PASQUALE baseline and follow-up assessments found better overall results than studies conducted in university hospitals in comparable settings such as Mali and Ethiopia [11, 12]. Baseline assessment found a knowledge score of 13.0 (out of 25) and a compliance of 23.7%. This percentage is elevated compared to Mali and Ethiopia (8.0 and 1.4% respectively). The high baseline compliance may be due to heightened awareness as a result of the Ebola outbreak: Several NGOs intervened during and after the outbreak by donating ABHR and by training HCWs [25]. Follow-up found a compliance of 71.5%, which is a threefold increase over baseline compliance. This follow-up compliance in Guinea was thus much higher than in Mali and Ethiopia with comparable timeframes (71.5% vs. 21.8 and 11.7%, respectively). A study from Rwanda demonstrated a similar follow-up compliance of 68.9%, but starting from a higher baseline level (34.1%) [26]. Applying the WHO methods [20], compliance was openly observed in FRH. As reported in previous studies, this open observation could lead to an overestimation of compliance (Hawthorne effect) [27]. This effect likely exists in comparable studies as well but could be weaker there than in FRH due to the observers’ longer presence in the other hospitals, which could have served to desensitize the HCWs to the observer presence. Success of the project indicated by the high post-intervention knowledge score and compliance may partially be attributed to the high motivation and involvement of the local team, including their dedicated coordinator. This commitment of the hospital and local authorities was shown in the independent organization of the “open day of patient safety” in the FRH. The introduction of the “Fascicle of the improved monitoring of hospitals” by the ministry of health in 2017 [21] could also have contributed to the high HH compliance in FRH. The limited size of the hospital, opportunities for direct communication among HCW and between HCW and hospital administration and leadership, as well as the prominence of the local ABHR production and HH campaign all play a potential role in leading to high compliance rates.

Compliance increased significantly across all five indications for HH. However, the indication “After touching patient surroundings” had consistently low compliance. HCWs may have had ambiguous understanding of what constitutes a patient surrounding, and difficulty compartmentalizing surroundings in the crowded and dynamic setting of a resource-limited facility where beds, personal space and belongings are often shared [11]. The indication “Before aseptic procedures” is considered to be one of the most critical moments for HAI prevention [28]. Compliance was alarmingly low for this indication prior to the intervention. Baseline assessment found many HCWs using gloves instead of taking the WHO recommended HH actions of washing or disinfecting hands [11, 12, 29]. This misuse of gloves is not unique to low resource settings [30, 31]. This critical indication “Before aseptic procedures” had the greatest increase in compliance post intervention, resulting in the highest compliance across all indications. Such strong improvement suggests the beneficial effect of training targeting previously identified shortcomings. A majority (89%) of baseline respondents reported having been trained in HH within the last 3 years, achieving a baseline knowledge score of 52.4%. The follow-up group achieved a significantly higher score of 75.6%. This increase indicates the importance of the multimodal strategy of PASQUALE which cornerstones an adaptive training and local production of ABHR.

Nurses and doctors had the highest number of HH opportunities and were also one of the highest scoring professional groups. Results across professional groups were consistent with previous findings from comparable settings in that medical doctors showed higher results in both knowledge and compliance [11, 12]. Interestingly, the opposite is observed in high income countries [32]. Perception of HCWs on HH was consistently positive and follow-up showed high acceptance of the intervention and awareness of its beneficial impact. Notable is the 75.3% gap between perceived versus observed compliance (99 and 23.7% respectively). This discrepancy highlights the potential overestimation of compliance through questionnaire surveys [33], and the importance of observations. There was a significant dip in self-evaluated compliance between baseline and follow-up which could mean that HCWs developed a more realistic perception of their compliance post-intervention.

The present study lacked capacity to assess healthcare-associated infection rates, reducing the ability to quantitatively measure intervention impact. However, WHO research shows that HH is the most effective tool to prevent HAIs [2].

Support to clarify language and question-type comprehension barriers within knowledge and perception questionnaires could possibly have influenced project results. This support was necessary to apply the WHO questionnaires within the study setting, where the form and language of such questionnaires is uncommon. The lack of a control group removed the possibility of adjusting for secular trends, but we did not identify any other competing intervention that may have influenced HH knowledge, perception or practice. Furthermore, multivariable logistic regression demonstrated that the association between intervention and compliance became even stronger after accounting for presumed and assessed confounders (crude odds ratio 6.67; adjusted odds ratio 16.40). This strengthens confidence that the improvements over time can be attributed, at least partially, to our intervention.

PASQUALE showed that the local ABHR production is a feasible method for providing self-sufficient supply of ABHR to regional hospitals such as the FRH. Costs of local production were within the WHO reported range and therefore considered to be cost-effective compared to commercially available products [22]. The observed fluctuation of production costs is subject to changing market prices of raw materials, e.g. of ethanol with is imported from neighboring countries such as Mali and Sierra Leone. These market fluctuations reflect the need in Guinea for reviving its own alcohol production capacities. Cost-effectiveness of local ABHR production is a driver for sustainability as the needed hospital budget for hygiene supplies can be reduced. Moreover, the local production team has expressed pride in their role to provide self-sufficient ABHR supply and remained motivated throughout. HCWs expressed their wish for local production in the baseline perception survey and rated it to be the key benefit of the intervention in follow-up survey. Consumption increased considerably, from 2.2 L to 28.0 L per month, over project time, hence closing the gap to the estimated minimum consumption needs of 40 L/month for the FRH. It would have been preferable to express ABHR consumption as quantity per patient-days, but these data are not recorded routinely by Faranah hospital. Since the hospital continued to function without major changes to its healthcare activities, the massive increase in ABHR per month is unlikely due to an increased average duration of hospitalization.

The participatory approach, such as FHR hygiene committee ownership of regular trainings and project-specific tasks, enhances prospects of sustainability. The extension of the project’s scope to include healthcare centers in the FHR coverage area furthers opportunities for knowledge exchange and entrenchment of regional HH knowledge and practice.

## Conclusion

The increase in ABHR consumption in conjunction with higher HH compliance highlights the potential for better patient safety, when three essential conditions are fulfilled in a hospital: access to supplies relevant for hospital hygiene (e.g. ABHR), knowledge of correct practice, and motivation through a good understanding of HH importance. Local ABHR production is a feasible and cost-effective method for providing self-sufficient supply of ABHR to regional hospitals like the FRH. The WHO HH Strategy is an adaptable and effective method to improve HH knowledge and compliance in a resource-limited setting like FRH.

## Supplementary information

**Additional file 1.**

## Data Availability

Main data generated or analysed during this study are included in this published article. Additional datasets used and/or analysed during the current study are available from the corresponding author on reasonable request.

## Abbreviations

ABHR: Alcohol-based hand-rub
CTEpi: *Centre de Traitement des Epidémies*
FRH: Faranah Regional Hospital
HH: Hand hygiene
HAI: Healthcare-associated infections
HCW: Healthcare workers
IQR: Interquartile range
OR: Odds ratio
WHO: World Health Organization

## References

[1] Health care-associated infections FACT SHEET. https://www.who.int/gpsc/country_work/gpsc_ccisc_fact_sheet_en.pdf. Accessed 26 Aug 2019.

[2] Guideline on hand hygiene in health care: a summary. https://www.who.int/gpsc/5may/tools/who_guidelines-handhygiene_summary.pdf. Accessed 26 Aug 2019.

[3] Allegranzi B, Bagheri Nejad S, Combescure C, Graafmans W, Attar H, Donaldson L (2011). Burden of endemic health-care-associated infection in developing countries: systematic review and meta-analysis. Lancet.

[4] Report on the burden of endemic health care-associated infection worldwide. https://apps.who.int/iris/bitstream/handle/10665/80135/9789241501507_eng.pdf;jsessionid=BC038FEA744C8E70AF89CC3F26C00E80?sequence=1. Accessed 4 Feb 2019.

[5] Keita A, Doumbouya N, Sow M, Konaté B, Dabo Y, Agbo Panzo D (2016). Prévalence des infections nosocomiales dans deux hôpitaux de Conakry (Guinée). Sante publique.

[6] Rosenthal VD, Maki DG, Graves N (2008). The international nosocomial infection control consortium (INICC): goals and objectives, description of surveillance methods, and operational activities. Am J Infect Control.

[7] Schreiber PW, Sax H, Wolfensberger A, Clack L, Kuster SP (2018). The preventable proportion of healthcare-associated infections 2005-2016: systematic review and meta-analysis. Infect Control Hosp Epidemiol.

[8] Health worker Ebola infections in Guinea, Liberia and Sierra Leone: a preliminary report. http://www.who.int/csr/resources/publications/ebola/health-worker-infections/en/. Accessed 26 Aug 2019.

[9] Pittet D, Allegranzi B, Sax H, Dharan S, Pessoa-Silva CL, Donaldson L (2006). Evidence-based model for hand transmission during patient care and the role of improved practices. Lancet Infect Dis.

[10] Allegranzi B, Pittet D (2009). Role of hand hygiene in healthcare-associated infection prevention. J Hosp Infect.

[11] Allegranzi B, Sax H, Bengaly L, Richet H, Minta DK, Chraiti MN (2010). Successful implementation of the World Health Organization hand hygiene improvement strategy in a referral hospital in Mali, Africa. Infect Control Hosp Epidemiol.

[12] Pfafflin F, Tufa TB, Getachew M, Nigussie T, Schonfeld A, Haussinger D (2017). Implementation of the WHO multimodal Hand Hygiene Improvement Strategy in a University Hospital in Central Ethiopia. Antimicrob Resist Infect Control.

[13] Schmitz K, Kempker RR, Tenna A, Stenehjem E, Abebe E, Tadesse L (2014). Effectiveness of a multimodal hand hygiene campaign and obstacles to success in Addis Ababa, Ethiopia. Antimicrob Resist Infect Control.

[14] Uneke CJ, Ndukwe CD, Oyibo PG, Nwakpu KO, Nnabu RC, Prasopa-Plaizier N (2014). Promotion of hand hygiene strengthening initiative in a Nigerian teaching hospital: implication for improved patient safety in low-income health facilities. Braz J Infect Dis.

[15] Guinea Human Development Indicators. http://hdr.undp.org/en/countries/profiles/GIN#. Accessed 24 Sept 2019.

[16] Pittet D, Allegranzi B, Storr J, Donaldson L (2006). 'Clean care is safer Care': the global patient safety challenge 2005-2006. Int J Infect Dis.

[17] Tools for evaluation and feedback. https://www.who.int/gpsc/5may/tools/evaluation_feedback/en/. Accessed 4 Feb 2019.

[18] Sax H, Allegranzi B, Uckay I, Larson E, Boyce J, Pittet D (2007). 'My five moments for hand hygiene': a user-centred design approach to understand, train, monitor and report hand hygiene. J Hosp Infect.

[19] Guidelines on hand hygiene in health care first global patient safety challenge clean care is safer care. https://apps.who.int/iris/bitstream/handle/10665/44102/9789241597906_eng.pdf;jsessionid=92EE378FF3106D946381D9F61338CB56?sequence=1. Accessed 26 Aug 2019. 23805438

[20] Sax H, Allegranzi B, Chraiti MN, Boyce J, Larson E, Pittet D (2009). The World Health Organization hand hygiene observation method. Am J Infect Control.

[21] Guinea M (2017). Fascicule du monitorage amélioré des hopitaux.

[22] Guide to local production: WHO-recommended handrub formulations. https://www.who.int/gpsc/5may/Guide_to_Local_Production.pdf?ua=1. Accessed 4 Feb 2019.

[23] Levy PLS (1991). Sampling of populations: methods and applications.

[24] Guide to implementation of the WHO mulitmodal hand hygiene improvement strategy. https://www.who.int/gpsc/5may/Guide_to_Implementation.pdf. Accessed 21 Aug 2019.

[25] Keita M, Camara AY, Traore F, Camara ME, Kpanamou A, Camara S (2018). Impact of infection prevention and control training on health facilities during the Ebola virus disease outbreak in Guinea. BMC Public Health.

[26] Holmen IC, Seneza C, Nyiranzayisaba B, Nyiringabo V, Bienfait M, Safdar N (2016). Improving hand hygiene practices in a rural Hospital in sub-Saharan Africa. Infect Control Hosp Epidemiol.

[27] Eckmanns T, Bessert J, Behnke M, Gastmeier P, Ruden H (2006). Compliance with antiseptic hand rub use in intensive care units: the Hawthorne effect. Infect Control Hosp Epidemiol.

[28] Scheithauer S, Batzer B, Molina CP, Widmer A (2013). P132: hand hygiene “before aseptic tasks”: a critical point even at a hematology and transplant ward. Antimicrob Resist Infect Control.

[29] Knowledge CA. Attitudes and Practices of Health Care Workers on Ebola in Hospital Towards Ebola Virus Disease, Conakry, Guinea, 2016. Cent Afr J Public Health. 2018;4(1):1–6.

[30] Wilson J, Prieto J, Singleton J, O'Connor V, Lynam S, Loveday H (2015). The misuse and overuse of non-sterile gloves: application of an audit tool to define the problem. J Infect Prev.

[31] Fuller C, Savage J, Besser S, Hayward A, Cookson B, Cooper B (2011). "the dirty hand in the latex glove": a study of hand hygiene compliance when gloves are worn. Infect Control Hosp Epidemiol.

[32] Erasmus V, Daha TJ, Brug H, Richardus JH, Behrendt MD, Vos MC (2010). Systematic review of studies on compliance with hand hygiene guidelines in hospital care. Infect Control Hosp Epidemiol.

[33] Watanabe T (2011). Discrepancy between self-reported and observed hand hygiene behavior in nurses and physicians. BMC Proc.

