# Supplementary material for: Implementation of the WHO hand hygiene strategy in Faranah regional hospital, Guinea
Source: Antimicrob Resist Infect Control. 2020 May 14;9:65. doi: 10.1186/s13756-020-00723-8 (PMC7227248; doi:10.1186/s13756-020-00723-8)
Supplement: Supplementary file 1 — Additional file 1. [file 13756_2020_723_MOESM1_ESM.doc]

Hand Hygiene Knowledge Questionnaire
for Health-Care Workers

| Period Number* |  |
| --- | --- |

- The knowledge required for this test is specifically transmitted through the WHO hand hygiene training material and you may find the questions more difficult if you did not participate in this training.
- Tick **only one answer** to each question.
- Please read the questions carefully before answering. Your answers will be kept confidential.
- **Short Glossary:**

**Alcohol-based handrub formulation:** an alcohol-containing preparation (liquid, gel or foam) designed for application to the hands to kill germs.

**Facility:** health-care setting where the survey is being carried out (e.g., hospital, ambulatory, long-term facility, etc).

**Handrubbing:** treatment of hands with an antiseptic handrub (alcohol-based formulation).

**Handwashing:** washing hands with plain or antimicrobial soap and water.

**Service:** a branch of a hospital staff that provides specified patient care.

**Ward:** a division, floor, or room of a hospital for a particular category or group of patients (it corresponds to the smallest segmentation of the health-care facility; one service can include multiple wards)**.**

| 1. Personal ID**: |  | 1. Date: |  |
| --- | --- | --- | --- |
|  |  |  |  |
| 1. Facility: |  | 1. Service**: |  |
|  |  |  |  |
| 1. Ward**: |  | 1. City:** |  |
|  |  |  |  |
| 1. Country**: |  |  |  |
|  |  |  |  |

1. Gender:  **Female**  **Male**
2. Age (years): **15-19**  **20-24** **25-29** **30-34** **35-39** **40-44** **45-49** **50-54** **55-59** **60+**
3. Profession***:  **Nurse**  **Auxiliary nurse**   **Midwife**  **Medical doctor**  **Resident**

Technician  Therapist Nurse student Medical student Other

* To be completed by the data manager.

** **Optional**, to be used if appropriate, according to the local needs and regulations.

*****Technicians**: radiologist, cardiology technician, operating room technician, laboratory technician

**Therapist**: physiotherapist, occupational therapist, audiologist, speech therapist

**Others**: dietician, dentist, social worker, etc.

Revised August 2009

1. Department (please select the department which best represents yours):

Internal medicine  Surgery  Intensive care unit  Mixed medical/surgical

Emergency unit  Obstetrics  Paediatrics  Long-term/rehabilitation

Outpatient clinic  Other

1. Did you receive formal training in hand hygiene in the last three years?  **Yes**  **No**
2. Do you routinely use an alcohol-based handrub for hand hygiene?  **Yes**  **No**
3. Which of the following is the main route of cross-transmission of potentially harmful germs between patients in a health-care facility? (*tick one answer only*)
4. Health-care workers’ hands when not clean
5. Air circulating in the hospital
6. Patients’ exposure to colonised surfaces (i.e., beds, chairs, tables, floors)
7. Sharing non-invasive objects (i.e., stethoscopes, pressure cuffs, etc.) between patients
8. What is the most frequent source of germs responsible for health care-associated infections?
   *(tick one answer only)*
9. The hospital’s water system
10. The hospital air
11. Germs already present on or within the patient
12. The hospital environment (surfaces)
13. Which of the following hand hygiene actions prevents transmission of germs *to the patient*?
14. Before touching a patient  Yes  No
15. Immediately after a risk of body fluid exposure  Yes  No
16. After exposure to the immediate surroundings of a patient  Yes  No
17. Immediately before a clean/aseptic procedure  Yes  No
18. Which of the following hand hygiene actions prevents transmission of germs *to the health-care worker*?
19. After touching a patient  Yes  No
20. Immediately after a risk of body fluid exposure  Yes  No
21. Immediately before a clean/aseptic procedure  Yes  No
22. After exposure to the immediate surroundings of a patient  Yes  No
23. Which of the following statements on alcohol-based handrub and handwashing with soap and
    water are true?
24. Handrubbing is more rapid for hand cleansing than handwashing  True  False
25. Handrubbing causes skin dryness more than handwashing  True  False
26. Handrubbing is more effective against germs than handwashing  True  False
27. Handwashing and handrubbing are recommended to be performed in sequence  True  False
28. What is the minimal time needed for alcohol-based handrub to kill most germs on your hands?
    *(tick one answer only)*
29. 20 seconds
30. 3 seconds
31. 1 minute
32. 10 seconds
33. Which type of hand hygiene method is required in the following situations?
34. Before palpation of the abdomen  Rubbing  Washing  None
35. Before giving an injection  Rubbing  Washing  None
36. After emptying a bedpan  Rubbing  Washing  None
37. After removing examination gloves  Rubbing  Washing  None
38. After making a patient's bed  Rubbing  Washing  None
39. After visible exposure to blood  Rubbing  Washing  None
40. Which of the following should be avoided, as associated with increased likelihood of colonisation of hands with harmful germs?
41. Wearing jewellery  Yes  No
42. Damaged skin  Yes  No
43. Artificial fingernails  Yes  No
44. Regular use of a hand cream  Yes  No

Thank you very much for your time!
